# Syndrome de pseudo-Foster Kennedy secondaire à une hypertension intracrânienne idiopathique: à propos d’un cas

**DOI:** 10.11604/pamj.2025.52.137.46908

**Published:** 2025-12-03

**Authors:** Asma Zaghdoudi, Maher Ben Said, Sameh Mbarek, Nada Sakli, Mohamed Habib Hedhiri, Olfa Blel, Mehdi Bouguerra, Wafa Ammari, Anis Mahmoud, Riadh Messaoud

**Affiliations:** 1 Hôpital Universitaire Tahar Sfar Mahdia, Mahdia, Tunisie,; 2 Hôpital Hedi Jaballah, Tozeur, Tunisie

**Keywords:** Hypertension intracrânienne, atrophie optique, œdème papillaire, ponction lombaire, cas clinique, Intracranial hypertension, optic atrophy, papilledema, lumbar puncture, case report

## Abstract

Le syndrome de pseudo-Foster Kennedy est une affection rare caractérisée par un œdème papillaire unilatéral avec une atrophie optique controlatérale en l'absence d'une masse intracrânienne causant une compression directe du nerf optique. Nous rapportons le cas d'une patiente âgée de 75 ans en surpoids (IMC 30 kg/m^2^), hypertendue mal équilibrée, qui a consulté pour une baisse de vision. L'interrogatoire a révélé la présence de céphalées évoluant depuis 2 mois. L'examen ophtalmologique a montré un œdème papillaire au niveau de l'œil droit et une atrophie du nerf optique au niveau de l'œil gauche. L'examen somatique a objectivé une hypertension artérielle mal équilibrée avec un pic hypertensif à 20/11. La patiente a bénéficié d'un examen neurologique ainsi que d'une imagerie cérébrale, qui étaient sans anomalies. Une ponction lombaire a été effectuée avec une résistance accrue à l'écoulement du liquide céphalorachidien (34 cmH2O). Les autres examens étaient normaux. Un syndrome de pseudo-Foster Kennedy dû à une HTIC idiopathique a été retenu. La patiente a été traitée avec l'acétazolamide par voie orale. L'évolution était marquée par une résolution complète de l'œdème papillaire et des céphalées avec une récupération progressive de l'acuité visuelle au niveau de l'œil droit. Une hypertension intracrânienne (HTIC) idiopathique représente une cause rare du syndrome de Pseudo-Foster Kennedy. Il n'a été rapporté que dans quelques cas dans la littérature. Il doit être évoqué devant ce syndrome pour préserver le pronostic vital et visuel.

## Abstract (en)

Pseudo-Foster Kennedy syndrome is a rare condition characterized by unilateral papilledema with contralateral optic atrophy in the absence of an intracranial mass causing direct compression of the optic nerve. We here report the case of a 75-year-old overweight female patient (BMI 30 kg/m^2^), with poorly controlled hypertension, who presented with decreased visual acuity. The patient reported headaches evolving over 2 months. Ophthalmologic examination showed papilledema in the right eye and optic nerve atrophy in the left eye. Physical examination revealed poorly controlled arterial hypertension with a peak blood pressure of 200/110 mmHg. The patient underwent neurological examination and brain imaging, both of which revealed no abnormalities. A lumbar puncture was performed and revealed increased resistance to CSF flow (34 cmH2O). Other tests were normal. The diagnosis of pseudo-Foster Kennedy syndrome secondary to idiopathic intracranial hypertension (IIH) was established. The patient was treated with oral acetazolamide. The evolution was marked by complete resolution of papilledema and headaches, with gradual recovery of visual acuity in the right eye. Idiopathic intracranial hypertension is a rare cause of pseudo-Foster Kennedy syndrome. Only a few cases have been reported in the literature. It should be considered when this syndrome is present in order to preserve both visual and vital prognosis.

## Introduction

Le syndrome de pseudo-Foster Kennedy est une affection rare. Il est caractérisé par un œdème papillaire unilatéral avec une atrophie optique controlatérale en l'absence d'une masse intracrânienne causant une compression directe du nerf optique [1]. La principale étiologie est une neuropathie optique ischémique antérieure non artéritique [2]. Le pseudo-syndrome de Foster-Kennedy secondaire à une hypertension intracrânienne idiopathique n'a été rapporté que dans quelques cas dans la littérature [3,4]. Nous rapportons un cas rare de syndrome de pseudo-Foster Kennedy chez une patiente présentant une hypertension intracrânienne (HTIC) idiopathique.

## Patient et observation

**Présentation du patient:** il s'agit d'une femme âgée de 75 ans qui a consulté pour une baisse de vision associée à des céphalées évoluant depuis 2 mois. Elle est en surpoids (IMC 30 kg/m^2^) et hypertendue, mal équilibrée.

**Figure 1:**
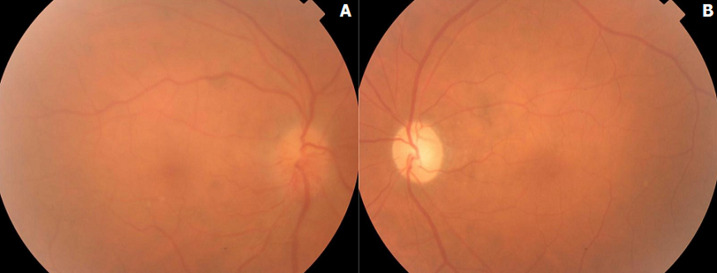
fond d'œil montrant un œdème papillaire au niveau de l'OD (A) et une atrophie optique au niveau de l'OG (B)

**Figure 2:**
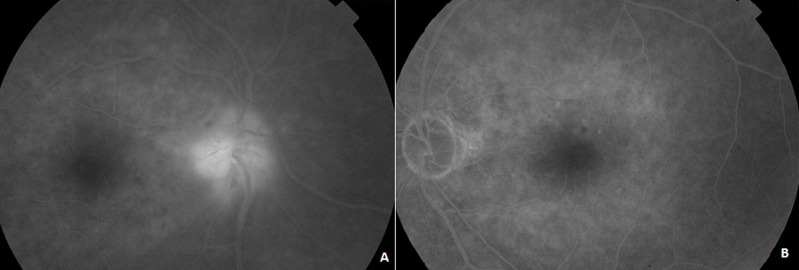
angiographie à la fluorescéine au temps tardif montrant une diffusion papillaire tardive au niveau de l'OD (A) et l'absence d'imprégnation papillaire au niveau de l'OG (B)

**Figure 3:**
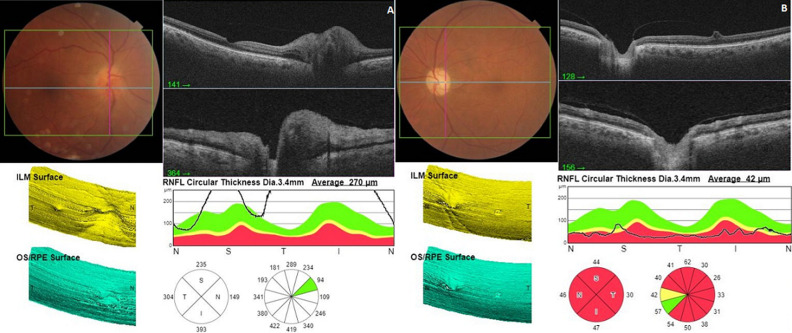
coupes d'OCT papillaire objectivant un épaississement diffus des couches RNFL au niveau de l'OD (A) et une altération diffuse des couches RNFL au niveau de l'OG (B)

**Résultats cliniques:** l'examen ophtalmologique a montré une acuité visuelle à 3/10, un RPM conservé et un œdème papillaire de grade 2 au niveau de l'œil droit (OD) (Figure 1, A). Au niveau de l'œil gauche (OG), l'acuité visuelle était limitée à compter des doigts avec le fond d'œil. On a trouvé une pâleur papillaire diffuse (Figure 1, B). Une angiographie à la fluorescéine a été pratiquée et a objectivé une hyperfluorescence papillaire au niveau de l'OD et une absence d'imprégnation papillaire au niveau de l'OG (Figure 2). La tomographie à cohérence optique papillaire a montré un épaississement des couches RNFL au niveau de l'OD et une altération profonde au niveau de l'OG (Figure 3).

**Démarche diagnostique:** l'examen somatique a révélé une hypertension artérielle mal équilibrée avec un pic hypertensif à 200/110 mmHg. Le bilan biologique, notamment la vitesse de sédimentation (VS) et la CRP (C réactive protéine), était correct. L'examen neurologique était sans anomalie. Une TDM orbito-cérébrale, effectuée en urgence, a révélé l'absence d'un processus expansif intracrânien. La patiente a bénéficié d'une mesure de la pression du liquide céphalo-rachidien par une ponction lombaire. La pression d'ouverture était élevée à 34 cm d'eau avec une composition normale. Le diagnostic d'un syndrome de pseudo-Foster Kennedy secondaire à une hypertension intracrânienne idiopathique a été retenu.

**Intervention thérapeutique:** elle a été traitée avec de l'acétazolamide par voie orale à la dose de 250 mg quatre fois par jour, avec une équilibration de sa pression artérielle.

**Suivi et résultats des interventions thérapeutiques:** l'évolution était marquée par une résolution complète de l'œdème papillaire et des céphalées sur une période de trois mois avec une récupération progressive de l'acuité visuelle au niveau de l'OD.

**Perspectives du patient:** la patiente était satisfaite du résultat du traitement avec une reprise progressive de ses activités habituelles.

**Consentement du patient:** la patiente a donné son consentement.

## Discussion

Avant l'avènement de la neuro-imagerie, le syndrome de Pseudo-Foster Kennedy (PFK) est initialement décrit suite à des craniotomies exploratoires d'un syndrome de Foster Kennedy sans cause compressive [5]. En dehors des neuropathies optiques ischémiques antérieures non artéritiques, plusieurs autres pathologies ont été rapportées comme responsables du syndrome de PFK. La névrite optique, le traumatisme et la syphilis représentent les plus fréquemment décrits [5]. D'autres causes rares, comme l'hypertension intracrânienne idiopathique (HTICI), ont été citées. L'HTICI est caractérisée par une pression intracrânienne élevée supérieure à 250 mmH2O, sans signe d'inflammation méningée, de lésion occupant l'espace ou de thrombose veineuse [6]. Elle se développe généralement chez les patients jeunes, de sexe féminin et obèses à l'âge de procréer [6]. Les céphalées et la diminution de la vision sont les symptômes les plus courants [6]. L'examen ophtalmologique objective fréquemment un œdème papillaire bilatéral [7]. L'asymétrie et l'unilatéralité de l'atteinte ont aussi été rapportées. Parmi les mécanismes décrits, on trouve la différence de diamètre entre les deux canaux optiques osseux, la compliance de la lame criblée et l'anatomie de la gaine du nerf optique. D'où s'installe une asymétrie de la résistance de l'écoulement du liquide céphalorachidien à partir des trabéculations dans l'espace sous-arachnoïdien de la gaine du nerf optique [8].

L'atrophie optique représente une complication redoutable [9]. Cette évolution dépend de la gravité et de la persistance de l'augmentation de la pression intracrânienne [5]. Ceci peut occasionner une perte de fibres nerveuses avec une perte de l'installation d'un gonflement papillaire [5]. Cette hypothèse peut expliquer la présence du syndrome de PFK associé à une HTICI. La pathogénie, couramment déterminée, consiste en un nerf optique atrophié ayant présenté initialement l'œdème papillaire le plus important, entraînant une atrophie optique, avec une préservation relative du nerf optique moins affecté, qui continue à manifester un œdème papillaire [5]. Une autre cause potentielle est admise, regroupant une atrophie optique dans un contexte d'une NAION superposée à l'hypertension artérielle non équilibrée ou une autre cause d'atrophie optique avant le développement de l'HTICI [5]. Pour cette patiente, nous admettons la première hypothèse vu qu'elle n'a pas rapporté la notion d'une baisse de la vision ancienne et qu’elle n'avait pas d'examen ophtalmologique auparavant étiquetant cette atteinte. L'évolution de la récupération visuelle dépend de la gravité et de la persistance de l'élévation de la pression intracrânienne. L'installation d'une atrophie optique est un critère d'irréversibilité. D'où l'importance d'un diagnostic précoce et d'un traitement par inhibiteurs de l'anhydrase carbonique pour réduire les niveaux de pression intracrânienne et pour éviter d'autres lésions au fil du temps [3].

## Conclusion

L'hypertension intracrânienne idiopathique doit être évoquée devant un syndrome de pseudo-Foster Kennedy. Devant cette entité oculaire, une prise en charge multidisciplinaire est primordiale afin de préserver le pronostic vital et visuel.

## References

[1] Mahjoub Y, Wan M, Subramaniam S (2023). Pearls & Oy-sters: Trigeminal Cystic Schwannoma Presenting With Foster Kennedy Syndrome, Sixth Nerve Palsy, and Focal Seizures. Neurology.

[2] Yoo KG, Chang JR (2024). Diabetic Papillopathy and Nonarteritic Anterior Ischemic Optic Neuropathy Presenting as Pseudo-Pseudo-Foster Kennedy Syndrome. J Neuroophthalmol.

[3] Visa Reñé N, Paredes Carmona F (2019). Pseudo-Foster Kennedy syndrome due to idiopathic intracranial hypertension. Arch Soc Esp Oftalmol (Engl Ed).

[4] Tirkey ER, Chandravanshi SL, Abdulrahman CY, Jain S (2025). Pseudo-foster kennedy syndrome due to idiopathic intracranial hypertension associated with empty Sella syndrome and hyperprolactinaemia: A rare case report. Delhi J Ophthalmol.

[5] Micieli JA, Al-Obthani M, Sundaram ANE (2014). Pseudo-Foster Kennedy syndrome due to idiopathic intracranial hypertension. Can J Ophthalmol.

[6] Mandura R, Khawjah D, Alharbi A, Arishi N (2023). Visual outcomes of idiopathic intracranial hypertension in a neuro-ophthalmology clinic in Jeddah, Saudi Arabia. Saudi J Ophthalmol.

[7] Horkovicová K, Cmelo J, Jurenova D, Furdova A (2020). Idiopathic intracranial hypertension with optic nerve edema - treatment options, case report. Neuro Endocrinol Lett.

[8] Swinkin E, Jabehdar Maralani P, Sundaram AN (2022). Unilateral Papilledema in Idiopathic Intracranial Hypertension: A Case Series. Can J Neurol Sci.

[9] Paramo R, Leishangthem L (2023). Optic atrophy secondary to minocycline-induced idiopathic intracranial hypertension. BMJ Case Rep.

